# Dialysis Access Dysfunction

**DOI:** 10.1155/2012/612025

**Published:** 2012-04-12

**Authors:** Alexander Yevzlin, Arif Asif, Anil K. Agarwal

**Affiliations:** ^1^Interventional Nephrology and Vascular Access Program, University of Wisconsin, 5148 MFCB 1685 Highland Avenue Madison, WI 53705, USA; ^2^University of Miami, Coral Gables, FL 33146, USA; ^3^Ohio State University, Columbus, OH 43210, USA

Vascular access failure (VAF) is the most common reason for hospitalization among hemodialysis (HD) patients. The economic burden of VAF is estimated to be greater than 1 billion dollars per year and continues to grow. The purpose of this special issue is to focus on recent advances in our understanding of dialysis access dysfunction.

Thanks in part to several national initiatives, the rate of arteriovenous fistula (AVF) placement continues to rise in the United States. AVF failure remains a major concern. Although the detection of early stenosis with preemptive correction prior to thrombosis seems to be a plausible option to prevent access failure, there is much debate, on the basis of of surveillance studies, as to whether early surveillance actually improves the longevity of an access system.

Evaluating the available information for surveillance, specifically the data for AVF stenosis and survival, is necessary to determine if surveillance is of any benefit. In an attempt to clarify ambiguities, one of the articles in this issue attempts to review the question: Does regular surveillance improve the long-term survival of arteriovenous fistulas?

Similarly, L. Kumbar et al., have contributed to this special issue with an evaluation of access surveillance outcomes. They state that although different techniques and methods are available for identifying access dysfunction, the scientific evidence for the optimal methodology is lacking. A small number of randomized controlled trials have evaluated the role of different surveillance techniques. The authors conclude that the limited randomized studies especially involving fistulae and small sample size of the published studies with conflicting results highlight the need for a larger multicentered randomized study with hard clinical end points to evaluate the optimal surveillance strategy for both fistulas and grafts.

Another important contribution is made by M. L. Zadeh et al. in this special issue. The authors observe that while native AVF is the recommended vascular access for HD, its failure to mature remains a major problem. The aim of their study was to determine the correlation between diameter and maturation of vessels in radiocephalic AVF. The authors performed a prospective cross-sectional study carried out during 2006-2007 on 96 hemodialysis patients from Hasheminejad Kidney Center. The maturation of fistula showed correlation with vein diameter, but no correlation was seen with the diameter of the arteries.

Inflammation is a problem for dialysis access as well as for ESRD patients' cardiovascular health. The contribution of the dialysis vascular access type to inflammation, however, remains largely undefined. This special issue contains a paper describing a prospective observational study in an incident HD population. C-reactive protein (CRP), interleukin-6 (IL-6), and interferon-*γ*-induced protein (IP-10) were measured before and at 6-time points after access placement for 1 year. A mixed effects model was performed to adjust for age, sex, race, coronary artery disease, diabetes mellitus, infections, access thrombosis, initiation of HD, and days after access surgery. In comparison to AVFs, the presence of a tunneled catheter (TC) was associated with significantly higher levels of CRP. Patients who initiate HD with a TC or an AVG have a heightened state of inflammation, which may contribute to the excess 90-day mortality after HD initiation.

These paper and several additional important contributions comprise this special issue on dialysis access. The guest editors hope that the readership of International Journal of Nephrology finds these contributions useful and enlightening.

*Alexander Yevzlin* *Arif Asif* *Anil K. Agarwal*

